# A perceptual pitch boundary in a non-human primate

**DOI:** 10.3389/fpsyg.2014.00998

**Published:** 2014-09-15

**Authors:** Olivier Joly, Simon Baumann, Colline Poirier, Roy D. Patterson, Alexander Thiele, Timothy D. Griffiths

**Affiliations:** ^1^Auditory Group, Institute of Neuroscience, Newcastle University, Newcastle upon TyneUK; ^2^Department of Experimental Psychology, MRC Cognition and Brain Sciences Unit, University of OxfordOxford, UK; ^3^Department of Physiology, Development and Neuroscience, University of Cambridge, CambridgeUK; ^4^University College LondonLondon, UK

**Keywords:** pitch discrimination, monkey model, psychophysics, auditory perception, harmonic sounds

## Abstract

Pitch is an auditory percept critical to the perception of music and speech, and for these harmonic sounds, pitch is closely related to the repetition rate of the acoustic wave. This paper reports a test of the assumption that non-human primates and especially rhesus monkeys perceive the pitch of these harmonic sounds much as humans do. A new procedure was developed to train macaques to discriminate the pitch of harmonic sounds and thereby demonstrate that the lower limit for pitch perception in macaques is close to 30 Hz, as it is in humans. Moreover, when the phases of successive harmonics are alternated to cause a pseudo-doubling of the repetition rate, the lower pitch boundary in macaques decreases substantially, as it does in humans. The results suggest that both species use neural firing times to discriminate pitch, at least for sounds with relatively low repetition rates.

## INTRODUCTION

For humans, pitch perception is important because pitch conveys prosody information in speech and melody information in music. It also conveys species information and affect in animal calls. For all of these environmental, multi-harmonic tones, the pitch value is closely related to the repetition rate of the acoustic wave. For humans, pitch discrimination is very accurate down to repetition rates of 50 Hz. Then, over the octave between 40 and 20 Hz, discrimination deteriorates rapidly, becoming statistically unreliable near 30 Hz. This “lower limit of melodic pitch” (Pressnitzer et al., 2001) corresponds to the lowest C on the piano keyboard. For humans, somewhere between 20 and 10 Hz, there is a distinct change in the quality of the percept (Guttman and Pruzansky, 1962; Warren and Bashford, 1981). It remains unclear whether non-human primates perceive pitch much as humans do. The purpose of this paper was to test this hypothesis by determining the lower limit of pitch in macaques. Previous work on macaques (Brosch et al., 2004) has suggested that they are very difficult to train on pitch tasks. Here we implement a new procedure to train macaques to discriminate the pitch of harmonic tones at low repetition rates which is successful after just weeks of training.

The harmonic tones produced by animals and musical instruments are pulsive; that is, they have a large peak factor because the excitation mechanism produces a regular sequence of acoustic pulses (Patterson et al., 2008). The spectra of these broadband, pulsive tones reveal a series of harmonics of the repetition rate and the dominant harmonics are all in cosine phase (Fletcher and Rossing, 1998). The pulsive nature of the excitation is preserved in the cochlea and it is argued that when the fundamental of the tone is relatively low, the auditory system computes the pitch using the time intervals between successive pulses in the internal representation of the sound (e.g., Patterson et al., 1995). One test of this hypothesis is to manipulate the phases of successive harmonics so that they alternate between 0 and π/2 radians. This does not alter the magnitude spectrum of the sound but it introduces a second peak within the period of the wave near the mid-point of the period. For humans, this manipulation decreases the lower limit of pitch substantially, presumably because the secondary peaks make it possible to measure time intervals accurately in waves with longer periods (lower repetition rates). Accordingly, the lower limit of pitch for macaques was measured with both cosine-phase tones and alternating-phase tones.

The lower limit of pitch in humans has been measured with a melody deviation task (Pressnitzer et al., 2001) and a traditional, forced-choice, pitch discrimination task (Krumbholz et al., 2000). The two paradigms provide comparable estimates of the lower limit of pitch. The pitch discrimination task of Krumbholz et al. (2000) is more appropriate for use with non-human primates, and was adapted here to provide a straightforward means of defining and measuring the lower limit of pitch in macaques.

## MATERIALS AND METHODS

### ANIMALS

Three male rhesus monkeys (*Macaca mulatta*) participated in this experiment. The animals M1, M2, and M3 (weighing 13, 14, and 18 kg) were 6, 5, and 10 years of age, respectively. Prior to this training, the animals had been trained to sit in a primate chair, to perform a visual detection task, and to manipulate the touch bar. Two of the animals had previous exposure to experimental auditory stimuli but in a passive visual fixation task. All experiments were carried out in accordance with the European Communities Council Directive RL 2010/63/EC, the US National Institutes of Health Guidelines for the Care and Use of Animals for Experimental Procedures and the UK Animals Scientific Procedures Act (PPL 60/4037) and were performed with great care to ensure the well-being of the animals.

### PARADIGM AND STAIRCASE PROCEDURE

**FIGURE 1:**
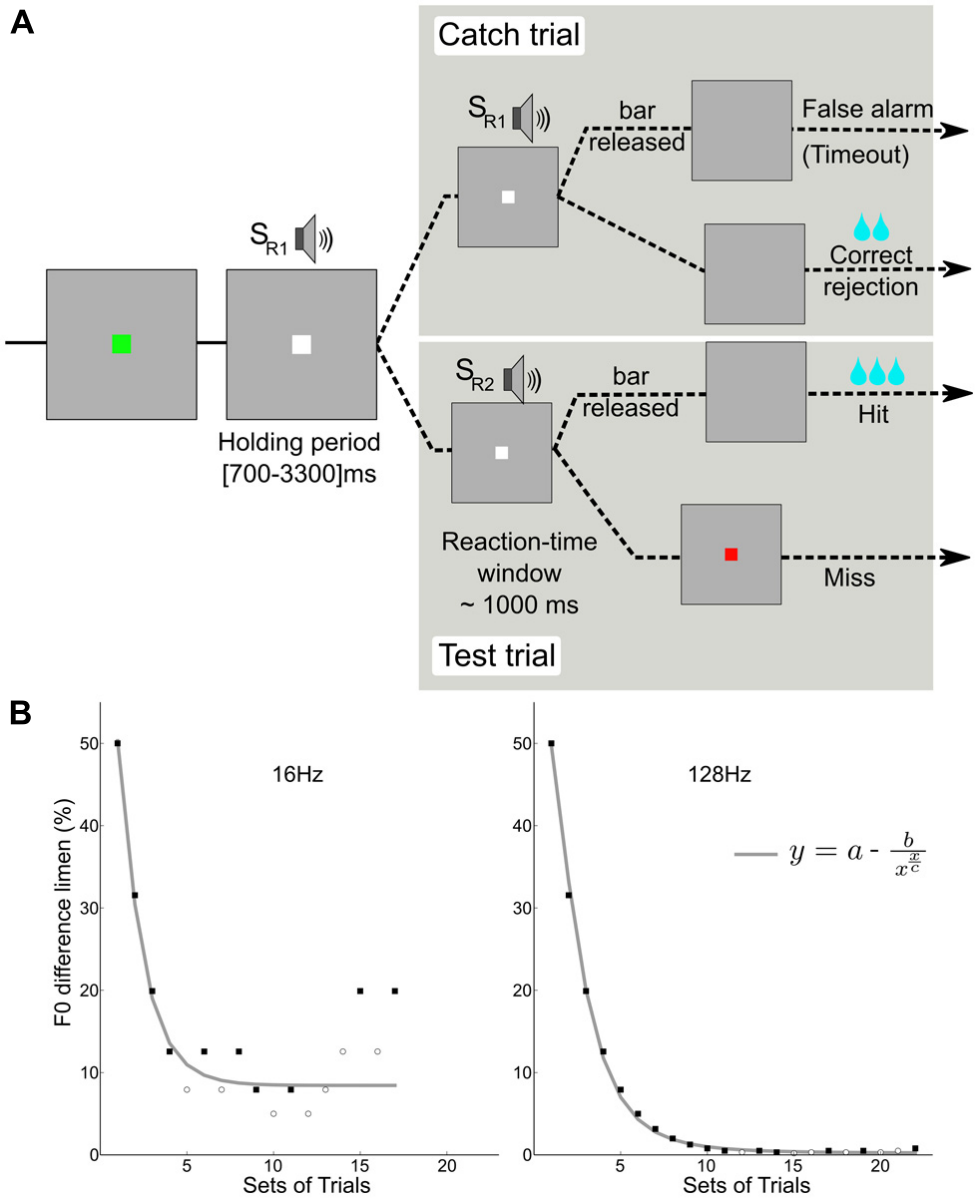
**Behavioral paradigm. (A)** A go/no-go task was used in which the monkey had to release a touch bar at each detection of a change in F0 during test trials (2/3 of the trials), and keep holding the bar during catch trials (1/3 of the trials). **(B)** Two adaptive tracks for subject M1; detection of a change in the pitch of two reference F0s (16 and 128 Hz). The staircase data were fitted with an asymptotic function using a standard least-squares algorithm. Filled squares and open circles represent hits and misses, respectively.

Animals were trained to release a touch bar at each detection of a change in the sound stimuli using a “go/no-go” procedure (**Figure 1A**). The monkey was installed in a primate chair in front of a screen and the sounds were played diotically through earphones. The experimental set-up was controlled with an in-house program, PrimatePy (Joly et al., 2014), written in the Python programming language; it was partly based on Psychopy (Peirce, 2007), a python-based psychophysics package. The fluid reward system and the touch bar from Crist Instrument were both controlled by the python software via a DAQ-Labjack (U3) USB device.

A trial (**Figure 1A**) began with an initiation interval during which a flashing green square indicated that a trial could be initiated by contacting the touch bar. Upon contact, the flashing green square became a steady white square and a holding period (700–3000 ms) began. During this period, a constantly repeating standard stimulus (SR1) was presented. Then a stimulus change interval (∼1 s) was presented during which a comparison sound SR2 with a higher F0 was presented. The monkeys responded with bar release to detected changes and were rewarded by a fluid reward when correct. Catch trials, in which the comparison sound was identical to the standard, were used to monitor when the animal was guessing. The animal was rewarded for not releasing during the catch trials; a response in the absence of change (false alarm) caused a time-out of about 2.6 s to be added to the inter-trial time (700 ms). Reward amount was increased with the progression of the staircase. The reward was higher for hits than for correct rejections.

**FIGURE 2:**
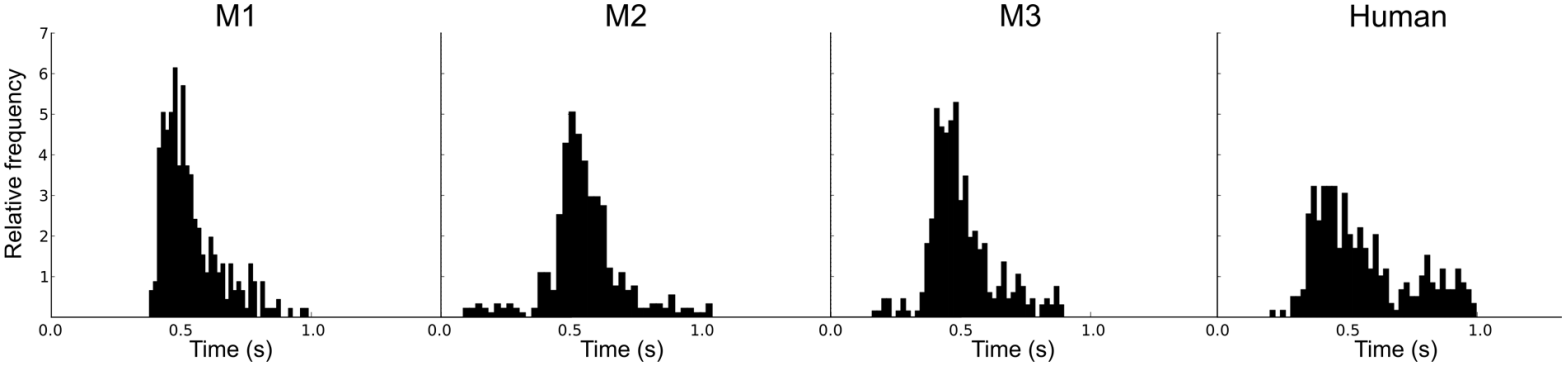
**Individual reaction time histograms showing the distribution of the reaction times for successful test trials (hits) separately for the three macaques, and a comparison distribution for a human**.

An adaptive staircase procedure from human psychophysics (Krumbholz et al., 2000) was modified for use with the macaques (Moore and Fallah, 2004). Blocks of two test trials and one catch trial were presented in random order: If the monkey successfully detected at least one of the F0 changes (50%) and successfully completed the catch trial (100%), the F0 was decreased. If the monkey failed to detect at least one change or failed the catch trial, the F0 was increased. This procedure rapidly brackets discrimination threshold; the combination of test trails and catch trials restricts variation in the response criteria of the monkeys. For each staircase, the procedure involved no fewer than 17 blocks and 8 reversals. In order to encourage their best performance, the reward size increased with each down step. **Figure 1B** shows examples of the adaptive tracks obtained for reference F0s of 16 and 128 Hz in one subject (M1). **Figure 2** shows the distribution of reaction times for the correct test trials (hits) for the three monkeys alongside one for a human; the attention and precision of the monkeys is clear.

The staircase of data was collected for each F0 value. The order of staircase presentation was randomly permuted within each series. For each F0 value, an estimate of the lower limit of pitch was obtained by fitting the asymptotic function $y=a-\frac{b}{x^{\left(\frac{x}{c}\right)}}$ to the data using a least-squares criterion. In the function, *y* is the F0 difference limen, x is the block number (set of trials), “*a*” is the asymptotic value, *c* is the block number at asymptote.

### STIMULI

The stimuli were cosine-phase harmonic tones and alternating-phase harmonic tones with fundamental frequencies, F0s, of 8, 16, 32, 64, or 128 Hz. Cosine-phase harmonic complexes are characterized by one click per period. Alternating-phase harmonic tones with the same F0 have the identical power spectrum but the phase alternates between 0 and π/2 radians as harmonic number increases, and this has the effect of inserting a secondary peak mid-way through the period of the wave. The harmonic complexes were composed of *N* = 8000/F0 harmonics with equal amplitudes. A low-pass filter with a cutoff frequency of 1 kHz and a slope of -6 dB/octave beyond the cutoff was applied to the stimuli (a finite-impulse-response Butterworth filter). The stimuli were switched on and off with cosine-squared gates with 8 ms rise/fall times. Within a trial, the duration of the standard stimulus (SR1) was randomly selected from a uniform distribution about 375 ms (+/-20%). The stimulus was repeated after a silent gap of 125 ms (+/-20%) until the presentation of the stimulus change (SR2), which consisted of a stimulus with a higher F0 (or an equal F0 on catch trials). The duration of the stimuli, the duration of the silent gap, and the duration of the holding period were all randomized to ensure that the animal could not simply rely on timing as a cue to bar release. The stimuli were computed with a sampling rate of 44.1 kHz with an in-house program written in Python.

## RESULTS

### THE PITCH BOUNDARY FOR COSINE PHASE TONES

**FIGURE 3:**
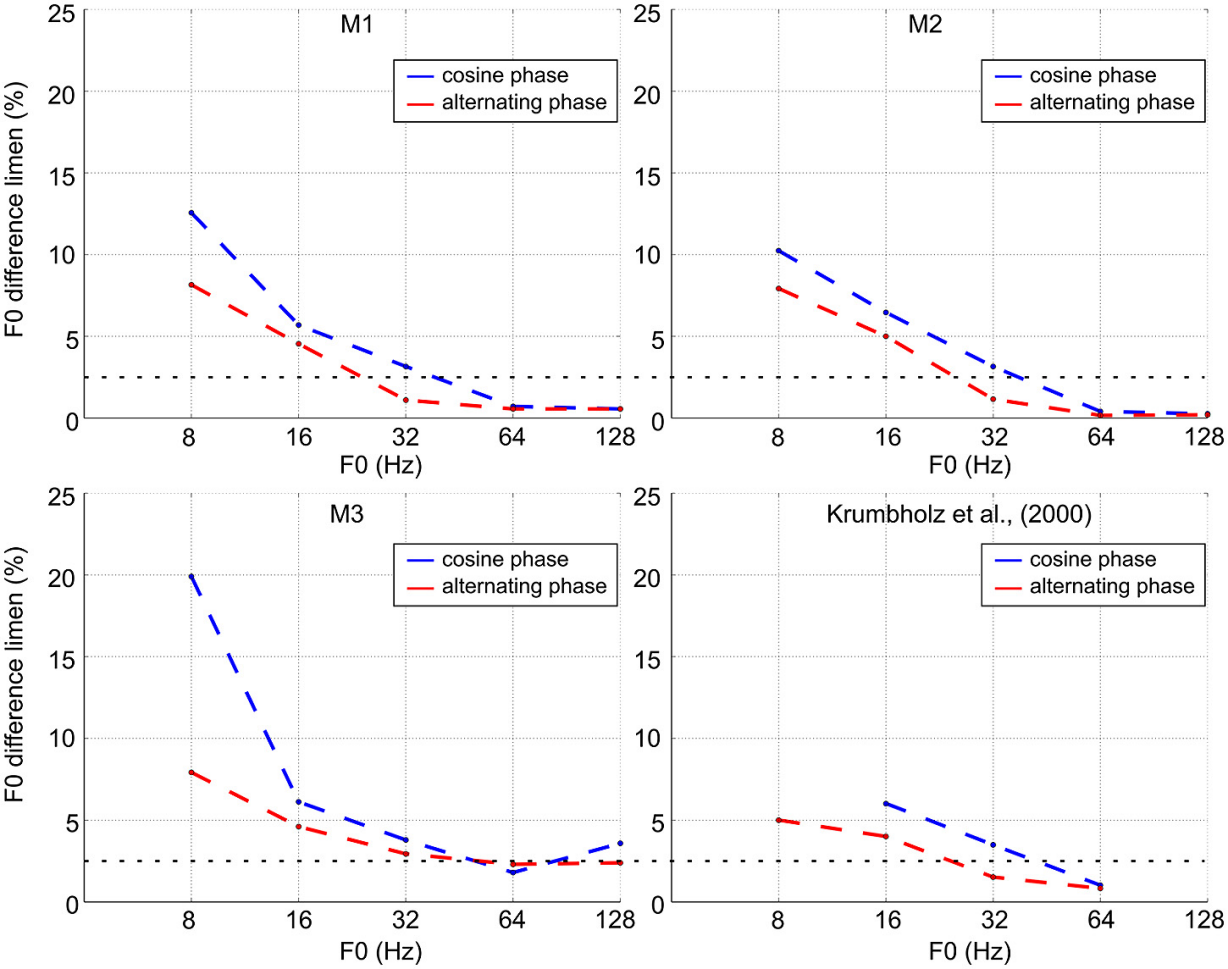
**F0 discrimination threshold as a function of F0 for cosine-phase and alternating-phase tones for the three macaques and a comparison set of average thresholds for humans (adapted from Krumbholz et al., 2000)**.

The blue lines in **Figure 3** show discrimination performance as a function of F0 for cosine-phase tones. The dashed horizontal line at 2.5% shows the threshold criterion used with humans (Krumbholz et al., 2000). At the higher values of F0 (64 and 128 Hz), the monkeys’ performance is similar to that of trained human listeners (difference limens below 2.5%). For two of the monkeys, the difference limen is less than 1% at these reference F0s; for the third monkey, it is about 2.5%. When the reference F0 is 32 Hz, the difference limen is just over 2.5% which is comparable to average human performance. Below 32 Hz, the difference limen increases rapidly toward 10%, just as it does with humans. For humans this increase is accompanied by the development of the ability to hear the individual pulses, and humans can discriminate a 10% change in pulse rate. This judgment is distinct from a pitch difference judgment. In this study, we illustrate two groups of case studies (macaques and humans) and demonstrated that in every macaque studied, the pitch difference limen increased as F0 decreased with a sudden change at the value, 32 Hz, which is the same value as for humans. Note that it remains unclear how to quantify statistically this cross-species similarity as it would involve defining the break point in the curve with its inherent variability across individuals and species.

### THE EFFECT OF WAVE SHAPE ON THE PITCH BOUNDARY

The red lines in **Figure 3** show discrimination performance for alternating-phase tones. For all three subjects, alternating phase produces better discrimination performance (the functions shift to the left below 64 Hz). Along the 2.5% criterion line, the lower limit of pitch for subjects 1 and 2 decreases by 0.67 and 0.58 octaves, respectively. Subject 3 shows a similar shift for a somewhat higher threshold criterion (around 4%).

## DISCUSSION

We have demonstrated that macaque monkeys can readily learn to discriminate small changes in the pitch of broadband harmonic sounds, provided the task has an encouraging balance of reward for correct performance and “time-outs” for errors. Previous studies of pitch discrimination with macaques used sequences of pure tones above 500 Hz (Brosch et al., 2004) and required tens of thousands of trials to learn a more difficult task involving short-term memory. Such studies may be affected by the fact that auditory working memory is limited in macaques (Scott et al., 2012). The go/no-go task of the current experiment is controlled by an adaptive threshold procedure that was developed to engage human listeners at the start of a run with easy trials, and then lead them to threshold on a track involving primarily positive trials and prompt increases in cue level whenever the listener makes an error. The very orderly tracking functions in **Figure 1B** show that the strategy also works well with macaques. Performance converges on F0 discrimination threshold rapidly for a wide range of F0 values.

In the region below 40 Hz or so, F0 discrimination deteriorates rapidly as it does with humans, indicating that the lower limit of pitch for macaques is very close to the lower limit for humans. When the phase spectrum of the harmonic complex is switched from cosine phase to alternating phase to double the rate of peaks in the waveform, all three monkeys demonstrate a dramatic reduction in threshold for the two lowest F0s (8 and 16 Hz). The same phenomenon occurs with humans (Krumbholz et al., 2000; Pressnitzer et al., 2001). Krumbholz et al. (2000) argued that, in the low frequency region, the pitch limit is determined by a temporal mechanism which analyses time intervals between peaks in the neural activity pattern flowing from the cochlea and, for some reason, the mechanism is limited to time intervals less than about 32 ms in duration. The fact that the lower limit of pitch for the macaque is close to 30 Hz with cosine-phase tones and considerably lower with alternating-phase tones suggests that they have a very similar neural mechanism, and that the limit on processing is not determined by the audiogram or other species specific aspects of hearing.

The macaque is considered to be a good model for human cortical organization with core and belt areas that are located in the superior temporal plane within the lateral fissure (Baumann et al., 2013). In macaques, there is an abrupt increase in the BOLD response (fMRI) in an area adjacent to A1 when the F0 of temporally regular sounds rises above 30 Hz (Griffiths and Hall, 2012). In humans, there is a similar increase in the BOLD response at the same value of F0 in a homologous area of human auditory cortex (Griffiths and Hall, 2012; **Figure 1**). The behavioral data suggest that both activations can be interpreted as pitch responses.

The measurement of the perceptual pitch boundary provides a practical means of locating pitch processing activity in macaque brains as well as human brains; the abrupt increase in activity as F0 rises above the lower limit of pitch is a *sine qua non* for the relevant neuronal mechanism.

## Conflict of Interest Statement

The authors declare that the research was conducted in the absence of any commercial or financial relationships that could be construed as a potential conflict of interest.
